# Adaptation of the Black Yeast *Wangiella dermatitidis* to Ionizing Radiation: Molecular and Cellular Mechanisms

**DOI:** 10.1371/journal.pone.0048674

**Published:** 2012-11-06

**Authors:** Kelly L. Robertson, Anahita Mostaghim, Christina A. Cuomo, Carissa M. Soto, Nikolai Lebedev, Robert F. Bailey, Zheng Wang

**Affiliations:** 1 Center for Bio/Molecular Science and Engineering, Naval Research Laboratory, Washington, D.C., United States of America; 2 Health Physics Section, Naval Research Laboratory, Washington, D.C., United States of America; 3 The Broad Institute of MIT and Harvard, Cambridge, Massachusetts, United States of America; 4 College of Science, George Mason University, Fairfax, Virginia, United States of America; University of Minnesota, United States of America

## Abstract

Observations of enhanced growth of melanized fungi under low-dose ionizing radiation in the laboratory and in the damaged Chernobyl nuclear reactor suggest they have adapted the ability to survive or even benefit from exposure to ionizing radiation. However, the cellular and molecular mechanism of fungal responses to such radiation remains poorly understood. Using the black yeast *Wangiella dermatitidis* as a model, we confirmed that ionizing radiation enhanced cell growth by increasing cell division and cell size. Using RNA-seq technology, we compared the transcriptomic profiles of the wild type and the melanin-deficient *wdpks1* mutant under irradiation and non-irradiation conditions. It was found that more than 3000 genes were differentially expressed when these two strains were constantly exposed to a low dose of ionizing radiation and that half were regulated at least two fold in either direction. Functional analysis indicated that many genes for amino acid and carbohydrate metabolism and cell cycle progression were down-regulated and that a number of antioxidant genes and genes affecting membrane fluidity were up-regulated in both irradiated strains. However, the expression of ribosomal biogenesis genes was significantly up-regulated in the irradiated wild-type strain but not in the irradiated *wdpks1* mutant, implying that melanin might help to contribute radiation energy for protein translation. Furthermore, we demonstrated that long-term exposure to low doses of radiation significantly increased survivability of both the wild-type and the *wdpks1* mutant, which was correlated with reduced levels of reactive oxygen species (ROS), increased production of carotenoid and induced expression of genes encoding translesion DNA synthesis. Our results represent the first functional genomic study of how melanized fungal cells respond to low dose ionizing radiation and provide clues for the identification of biological processes, molecular pathways and individual genes regulated by radiation.

## Introduction

Fungi are generally highly radioresistant when inhabiting environments characterized by elevated levels of ionizing radiation [1], [2]. Most intriguingly, the walls and cooling water of the damaged nuclear reactor at Chernobyl, which are constantly exposed to ionizing radiation, harbor large of amounts of microorganisms, including fungal species [3], [4]. Furthermore, Zhdanova et al. reported that beta and gamma radiation promoted directional growth of fungi isolated from the Chernobyl accident site towards the source of ionizing radiation [5]. They termed this attraction of organisms to radiation “radiotropism”. Therefore, in contrast to the general view that radiation is detrimental to life, it is thought that fungi have adapted the ability to survive or even benefit from exposure to ionizing radiation. To support this notion, Dadachova et al. demonstrated that ionizing radiation could enhance the growth of melanized fungi and change the electronic properties of melanin [6]. These investigators studied the interaction of ionizing radiation with three fungal species: *Cryptococcus neoformans*, which can grow into a melanized form when supplied certain exogenous substrates, and two intrinsically melanized species *Wangiella dermatitidis* and *Cladosporium sphaerospermum* with the latter being the predominant species colonizing in the damaged reactor at Chernobyl. Although constantly exposed to ionizing radiation approximately 500 times higher than background, those melanized fungal cells under nutrient limited conditions grew significantly faster as measured by higher colony forming units (CFU), more dry weight biomass and greater incorporation of ^14^C-acetate than non-irradiated cells or irradiated melanin defective mutants.

The finding that melanized fungal growth was enhanced by ionizing radiation was surprising. However, the underlying mechanisms that govern the molecular response of fungal cells to low doses of ionizing radiation during long-term exposure remain poorly understood, even though in the functional genomics era, evaluation of the global expression profiles is increasingly used to determine the genetic responses to environmental stresses. Currently, genomics studies of ionizing radiation effects on fungi are mainly performed with two model organisms, the budding yeast *Saccharomyces cerevisiae* and fission yeast *Schizosaccharomyces pombe* [7]–[10]. Those studies suggest that complicated networks of regulatory pathways are involved in coordinating gene expression responses in the presence of ionizing radiation. The majority of regulated genes were found to be associated with DNA damage checkpoints, cell cycle events and environmental stress responses. The radiation responses, however, were examined during recovery from acute exposure to lethal doses of ionizing radiation (IR). Therefore, gene expression measurements from those studies are likely different than those following exposure to the long-term low doses of ionizing radiation.

In this study, we used the black yeast *W. dermatitidis* as a model to investigate the molecular and cellular responses to chronic and low dose ionizing radiation. *W. dermatitidis*, also called *Exophiala dermatitidis*, is one of many species of the Fungi Imperfecti, which are darkly pigmented (dematiaceous) owing to the deposition of 1,8-dihydroxynaphthalene (1,8-DHN) melanin in their cell walls [11], [12]. This fungus was employed because it has been extensively studied physiologically, biochemically and most recently genetically, and is now considered to be the best model for more than 100 other dematiaceous agents of mycoses [13], [14]. In addition, because *W. dermatitidis* exists predominantly as a budding yeast form in vitro, it can be easily cultured using standard single cell procedures and also manipulated to undergo various morphological transitions to produce isotropically enlarged yeast, multicellular forms and various types of hyphae [15]. Furthermore, *W. dermatitidis* and other related species have been characterized as having remarkable thermotolerance, halotolerance and pH tolerance [16], the combination of which are rarely observed in fungi and attest to its extremophilic nature. Most importantly, *W. dermatitidis* was previously used as one of three model organisms to demonstrate enhanced growth induced by ionizing radiation [6]. These attributes together with its increasing molecular tractability, make *W. dermatitidis* an excellent system for studies of fungal growth during interaction with ionizing radiation.

Recently, the genome of *W. dermatitidis* was completely sequenced as a part of the Broad Institute Fungal Genome Initiative (http://www.broadinstitute.org/annotation/genome/Black_Yeasts/MultiHome.html), which is facilitating a systematic analysis of gene expression responses. Here we report the result of a transcriptomic study of gene expression responses to low dose ionizing radiation in *W. dermatitidis*, by using RNA-Seq technology [17]. Our results confirmed that low doses of ionizing radiation enhanced the growth of *W. dermatitidis* by altering its cell cycle progression. Comparison of the global gene expression profiles of the wild type and the melanin defective mutant *wdpks1* in irradiation and non-irradiation conditions identified more than 3000 differentially expressed genes, many of which are associated with protein translation, cell cycle, metabolism and stress responses. We also identified a number genes or gene clusters whose protein products participate in DNA translesion synthesis and antioxidation, which explains the increased survivability of fungal cells in the presence of low dose ionizing radiation. However, the transcriptomic data did not indicate that melanin played a significant role in the enhancement of cell growth except that it up-regulated expression of ribosomal biogenesis and transporter genes. Together, our results represent the first functional genomic study of how melanized fungal cells respond to low doses of ionizing radiation. This suggests future directions for the investigations of biological processes, signal transduction pathways and signature genes regulated by such radiation.

## Materials and Methods

### Growth and Irradiation Conditions for W. dermatitidis

The wild type strain of *W. dermatitidis* 8656 (ATCC 34100, *Exophiala dermatitidis* CBS 525.76) and the *wdpks1* mutant with a disrupted polyketide synthase 1 gene were obtained from Dr. Paul J. Szaniszlo (The University of Texas at Austin). These strains were cultured using the modified procedures from the previous publication [6]: single colonies from YPD (2% peptone, 1% Bacto Yeast extract and 2% dextrose) agar plates were inoculated into 5 ml YPD broth and cultured at 25°C with shaking at 200 rpm for 48 hours. Cell cultures were transferred (1∶100) in 5 ml YPD and grown with shaking at 37°C for 48 hours, and then diluted (1∶100) into 10 ml YPD and grown for another 24 hours. Cells with yeast forms were transferred into 20 ml defined minimal medium (3 g/L NaNO_3_, 1 g/L K_2_PHO_4_, 1 g/L MgSO_4_.7H_2_O, 0.5 g/L KCl, 0.003 g/L thiamine, 5.3 g/L NH_4_Cl, 120 mg/L sucrose, pH6.5) to 10^7^ cells/ml and cultured with shaking at 37°C for 24 hours. Then cells were collected, washed and diluted to 5x10^5^ cells/ml in the defined minimal medium. One ml or 20 ml cell aliquots in tubes were incubated in 37°C heatblocks without shaking in the dark. One group was placed in normal conditions without irradiation and another group was placed in the field of a ^137^Caesium with dose rate of 0.03 Rad (R)/hr (1 Gy = 100 Rad). At each time point, cell growth from triplicate samples was determined with either the automatic cell counter Cellometer Auto X4 (Nexcelom Bioscience LLC, Lawrence, MA) or by CFU counts.

### Preparation of RNA and RNA-seq

Duplicate biological samples from each condition were collected for RNA-seq experiments (Figure S1A). RNA-seq experiments were conducted at Genomics Resource Core Facility at Weill Cornell Medical College (New York, NY). Total RNA was extracted from non-irradiated or irradiated cultures of *W. dermatitidis* 8656 and *wdpks1* mutant with RiboPure™-Yeast Kit (Invitrogen, Carlsbad, CA). Enrichment of poly(A) RNA, synthesis of cDNA and construction of cDNA libraries were processed using the protocol provided by Illumina Inc. (San Diego, CA). Libraries were sequenced from 50 cycles on an Illumina HiSeq2000 Sequencer and at least 20 million single reads were generated from each sample.

### Data Analysis

Illumina reads were aligned to the predicted *W. dermatitidis* transcripts using Bowtie [18], and the number of reads mapping to each transcript were estimated using RSEM [19]. Transcripts differentially expressed between any two conditions were identified using EdgeR [20]. Functional categories of differentially expressed genes were annotated with the KEGG database [21]. Transcription modules (ribosomal biogenesis, cell cycle and amino acid biosynthesis) in *W. dermatitidis* were identified by comparing with those described in *S. cerevisiae* [22] with the BLAST algorithm.

### Quantitative Reverse Transcription PCR

Real-time reverse transcription PCR assays were conducted using iScript™ One-Step RT-PCR Kit with SYBR Green (Bio-Rad, Hercules, CA). 50 ng of total RNA from two biological samples were run in duplicate on an iCycler (BioRad Laboratories, Hercules, CA, USA). PCR primers (Table S1) were designed using Primer3 online software (v. 0.4.0) (http://frodo.wi.mit.edu/). Relative quantities of transcript were determined using the 2^−▵▵Ct^ formula. ▵Ct is the difference in Ct of the gene of interest and Ct of the normalizer gene, and ▵▵Ct is the difference in ▵Ct from the irradiated sample and ▵Ct from the non-irradiated sample. *CDC42* gene of *W. dermatitidis* was used to normalize expression levels of genes of interest because its transcript was constant in all conditions in this study.

### Measurement of Cell Sizes

Three ml of cell culture (∼3x10^7^ cells in total) grown with or without irradiation for 48 hours were collected and fixed with 3% paraformaldehyde at room temperature for 1 hour. Fixed cells were placed in 1 cm path-length cuvettes and cell sizes were determined using a ZetaPALS DLS system (Brookhaven Instruments Corporation) equipped with a red (658 nm) laser at a 90° angle. The Multimodal Size Distribution software processes data automatically based on a non-negative least squares (NNLS) analysis method [23] and all sizes reported here were based on volume weighed sizes. Ten measurements were taken per sample at room temperature.

### Fluorescence Activated Cell Sorting (FACS) and Flow Cytometry Measurements of DNA Content and Intracellular Reactive Oxygen Species (ROS)

For sorting cells into un-budded and budded populations, a JSAN FACS was utilized. Samples were sorted based on forward and side-scatter and the presence of un-budded and budded cells was confirmed using microscopy. The cells were concentrated to 5x10^5^ cells/ml and grown in the dark or under ionizing radiation (IR). Following IR treatment, 10^6^ cells/sample were centrifuged for 2 minutes and fixed with 70% ethanol overnight. The following day, the cells were washed twice with 1X PBS and either used immediately or stored at 4°C. The cells were then treated with 2mg/mL RNase A in 1X PBS for 6 hours at 37°C. Propidium iodide (PI) was added directly to the RNase A solution to a final concentration of 2.5 µM. Samples were sonicated prior to analysis with an AccuriC6 flow cytometer equipped with a 488 nM excitation laser and standard PI filters.

For measurement of intracellular ROS, cells were washed once with 1X PBS and resuspended in 20 µM dihydroethidium (Sigma, St. Louis) for 30 minutes at 30°C, cooled briefly on ice and washed twice with 1X PBS. The samples were resuspended in PBS and analyzed on an AccuriC6 cytometer equipped with a 488 nM excitation laser and a standard PE filter. For each sample analyzed by flow cytometry, 5 x 10^4^ events were collected in a gate corresponding with the cell population.

### Measurement of Carotenoid from Single Cells

Carotenoids were analyzed with Raman microspectroscopy and the Raman spectra of individual cells adsorbed on gold substrate were analyzed. The measurements were performed with an inVia Renishaw Raman microscope [http://www.renishaw.com]. The selective Resonant Raman (RR) excitation was used with 488 nm laser light that is within the main absorption bands of many carotenoids [24]. The spectra were recorded in the Stokes region (200–1800 cm^−1^) in a single scan with laser power 6 mW. Each scan was performed on a fresh cell and the spectra of each cell were analyzed individually. After subtraction background by polynomial fitting (usually 5-meres), the spectra were smoothed by binomial averaging over 9–15 points.

## Results

### Low Dose Ionizing Radiation Increases W. dermatitidis Growth Rate and Cell Size

**Figure 1:**
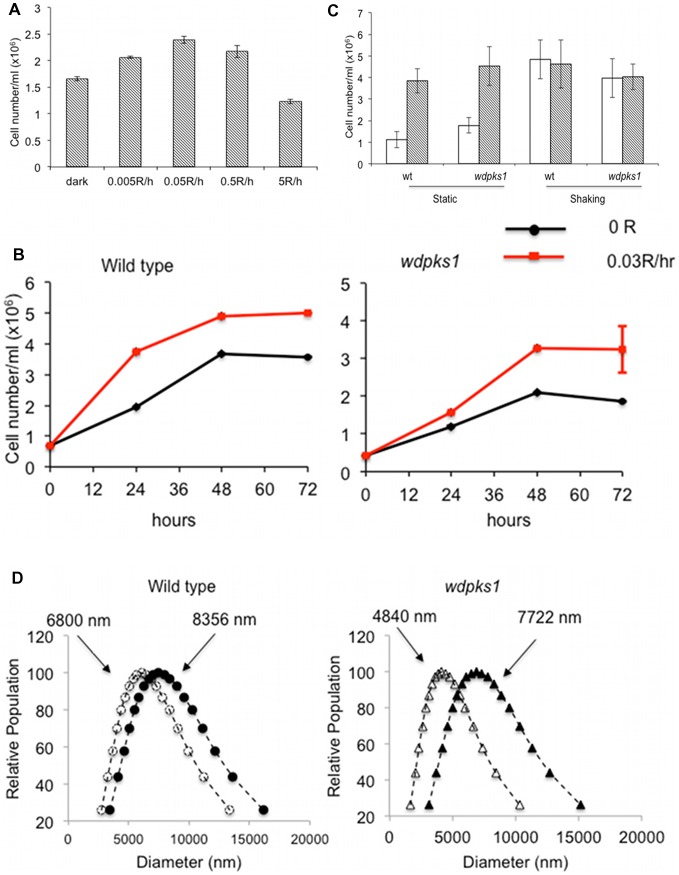
Effects of low doses of ionizing radiation on the growth rates and sizes of *W. dermatitidis* cells. (A) Effect of different doses of ionizing radiation on the growth of *W. dermatitidis* 8656 at 24 hr. (B) Growth comparison of non-irradiated and irradiated cells of *W. dermatitidis* 8656 and *wdpks1*. Cells were irradiated with 0.03R/hr of ionizing radiation. (C) Growth conditions (static and shaking) affect the effects of ionizing radiation on growth of *W. dermatitidis* 8656 and *wdpks1* at 48 hr. Non-irradiation (open bar), irradiation (0.03R/hr) (hatched bar). (D) Cell sizes were increased under ionizing radiation. The wild type (circle) and the *wdpks1* mutant (triangle) were grown in minimal media without (open) and with (solid) irradiation for 48 hr. Cell diameters in 3 ml cultures were measured with DLS. The mean cell diameter of each sample is shown at the top of the curve.

The effects of low dose ionizing radiation on the growth of the black yeast *W. dermatitidis* were first examined by irradiating the wild type cells with a variety of doses ranging from 0.005R/hr to 5R/hr. After 24 hours of exposure, three of four ionizing radiation doses (0.005R/hr, 0.05R and 0.5R/hr) resulted in the production of at least 30% more cells than were produced by the non-radiation control, as measured by cell numbers (Figure 1A). The number of cells was increased at 0.005R/hr and even more so at 0.05R/hr, but was inhibited at 5R/hr presumably due to the fungicidal effect of the higher dose. To further confirm the ionizing radiation effects on the wild type of *W. dermatitidis* and also on the melanin defective mutant *wdpks1* [25], we next constantly exposed these two strains to a dose of 0.03R/hr, which was equivalent to the radiation dose at the main gate of Fukushima Daiichi Nuclear Power Station in August of 2011 [26]. As expected, the irradiated wild-type cells grew significantly more than the non-irradiated control over 3 days (Figure 1B), which agreed with the result reported by Dadachova et al [6]. However, the increased cell number was also observed when the albino *wdpks1* mutant was irradiated, but the difference was more pronounced when cells entered the stationary phase. This trend was consistent when additional growth experiments were conducted under 0.2R/hr and 2R/hr doses of radiation (Figure S2). It was noted that the enhanced growth was only observed when cells were grown in minimal media and under static conditions. When cells were grown in rich media (data not shown) or liquid cell cultures were constantly shaken, the enhanced growth under ionizing radiation was abrogated (Figure 1C). These results suggest that rich nutrients, oxygen or a dynamic growth environment, which all benefit cell growth, would eliminate the effect of ionizing radiation.

In addition to cell numbers, we also examined cell sizes for a response to ionizing radiation. Cell sizes from 3 ml cell cultures with approximately 3x10^7^ total cells were determined using a dynamic light scattering system (Figure 1D). This revealed that irradiated cells of both the wild type and *wdpks1* strains were generally larger than non-irradiated cells. Because the cell wall of *wdpks1* is nearly 40% thinner than that of the wild type and absence of a polymerized melanin layer results in less rigidity of cell structure [27], it was not surprising to observe that *wdpks1* cells had relatively smaller sizes but greater degree of size expansion than the wild type.

**Figure 2:**
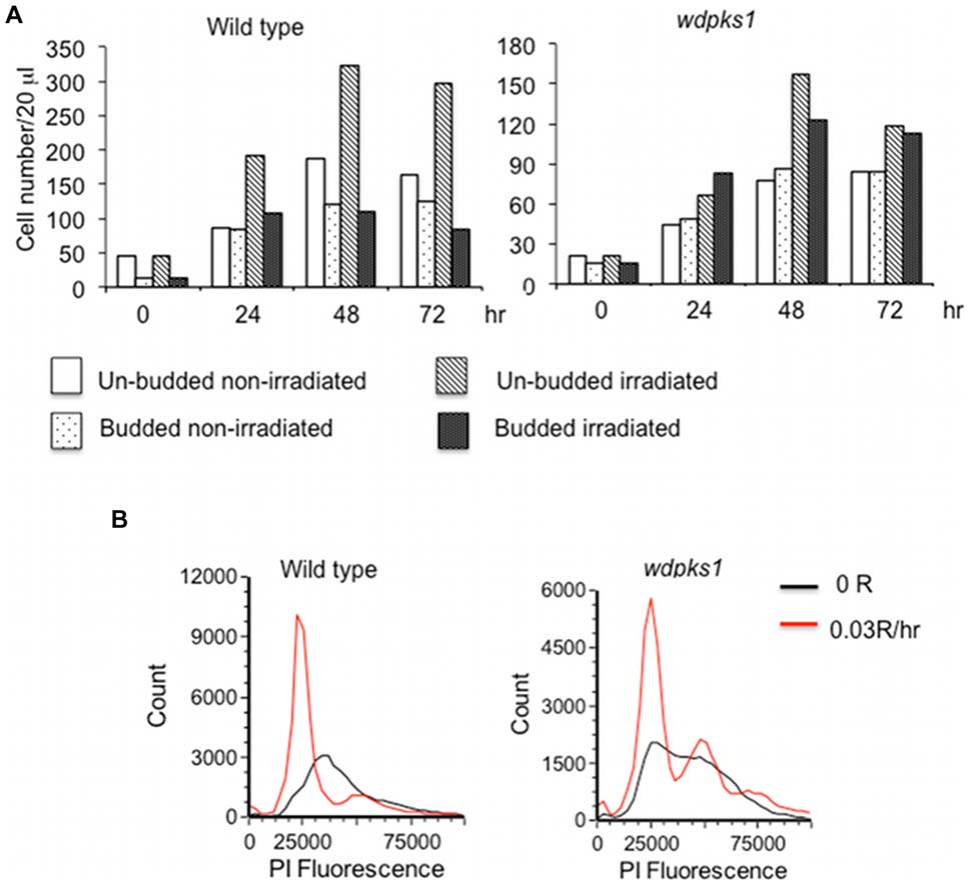
Effect of low dose of ionizing radiation on morphologies of *W. dermatitidis* cells. (A) Comparison of numbers of un-budded and budded cells in non-irradiated and irradiated (0.03R/hr) cultures of the wild type and the *wdpks1* mutant at four time points from Figure 1B. Cell number was counted from 20 µl cell culture with the automatic cell counter and un-budded and budded cells were manually recorded from cell counting images. (B) Flow cytometry analysis of DNA content of non-irradiated and irradiated cells (left: wild type; right: *wdpks1*) at 48 hr.

It was noted that there were larger numbers of un-budded cells in the irradiated cell samples than in the non-irradiated samples, which prompted us to examine the cell morphologies. At each time point depicted in Figure 1B, we compared the number of un-budded and budded cells in 20 µl of cell culture from each sample. Figure 2A showed that most of the enhanced growth of the wild type cells under 0.03R/hr radiation was due to the increased number of in un-budded cells in the culture. In addition, it appeared that deletion of the *WdPKS1* gene not only resulted in the albino phenotype but also affected cell morphology and produced clumpy and multiple budded cells. Although the effect of ionizing radiation on morphology of *wdpks1* mutant was not as pronounced as that induced in the wild type, it still tended to increase the number of un-budded cells (Figure 2A). We then employed flow cytometry to measure the DNA content of non-irradiated and irradiated cells. Consistent with the morphologies, irradiated cells from both the wild type and *wdpks1* strains predominately had G1 content of DNA (Figure 2B), which suggested that irradiated cells were arrested in the G1 phase of the yeast cycle.

### More than 3000 Genes are Differentially Regulated by Low Dose Ionizing Radiation

**Figure 3:**
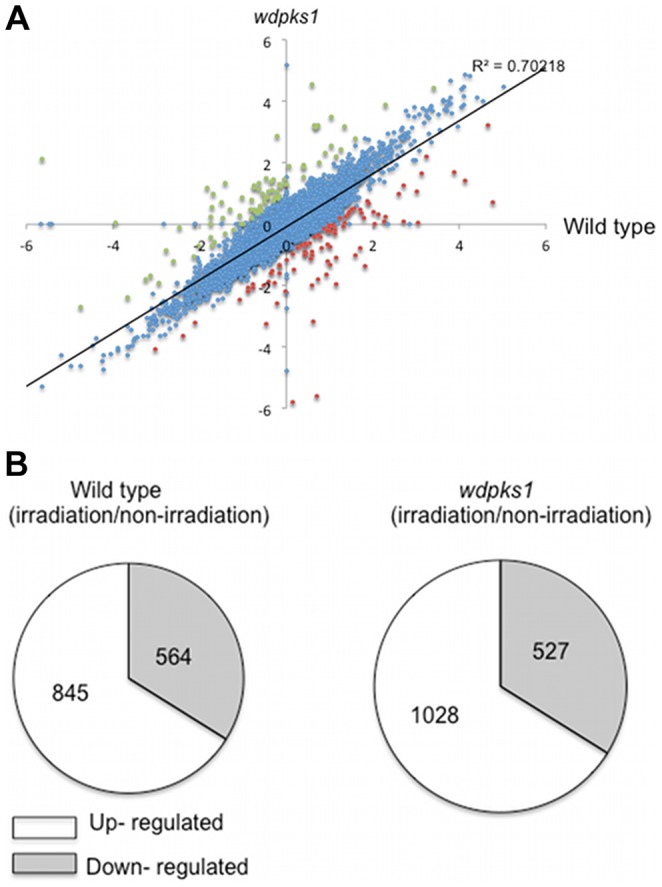
Transcriptomic analysis of gene expression responses to low dose ionizing radiation in *W. dermatitidis*. (A) Scatter plots of RNA-seq data for ∼9500 genes obtained from the wild type and *wdpks1* mutant. Each blue point represents the log2 change resulting from ionizing radiation in levels of the transcript for a single gene for the wild type in the horizontal dimension and the log2 change of the transcript for that gene in *wdpks1* strain in the vertical dimension. Red and green points are outliers representing more significant changes in either strain. (B) Identification of differentially expressed genes with >2 fold changes (FDR <0.001) in response to the low dose of ionizing radiation in the wild type and the *wdpsk1* mutant.

To investigate the molecular mechanism of how *W. dermatitidis* increases its growth and adapts to low dose ionizing radiation, we used RNA-seq technology to examine gene expression of the wild type and *wdpks1* mutant cells grown either under irradiation or non-irradiation conditions (Figure S1A). In the irradiation experiments, cells were constantly exposed to a 0.03R/hr dose for 48 hr, at which time cells were entering stationary phase. Differential expression analyses were first compared between the duplicate samples and no differentially expressed genes were identified (Figure S1B), demonstrating the low noise between replicate samples. Meanwhile, differential gene expression affected by radiation between the wild type and the *wdpks1* mutant was generally correlated as shown by the scatter plot in Figure 3A. A subsequent pair-wise, comparison analysis by using a false discovery rate (FDR) of 0.001 revealed that, in the wild-type strain, 3009 genes were differentially expressed in the irradiated cells relative to the non-irradiated cells, whereas in the *wdpks1* mutant, 3196 genes were differentially expressed under irradiation. Those differentially expressed genes accounted for ∼30% of 9562 annotated genes in the *W. dermatitidis* genome. To determine which of the genes were significantly affected by the low dose of ionizing radiation, we identified genes whose transcripts were changed by the radiation greater than 2 fold in the wild type and the *wdpks1* mutant (FDR <0.001). The result showed that 1409 (564 induced and 845 repressed) and 1555 (527 induced and 1028 repressed) genes were regulated by radiation in the wild type and *wdpks1* mutant, respectively (Figure 3B). Of the regulated genes, the majority (1148) overlapped in the two datasets. Meanwhile, 24 genes whose transcripts were regulated more than 2 fold in the wild type strain were chosen for verification with quantitative RT-PCR. Transcript changes measured with RNA-seq and qRT-PCR were generally in the same trend as shown in Table 1. Furthermore, about 30% of those regulated genes were assigned to functional classes by KEGG analysis. Overall, the wild type and *wdpks1* mutant shared similar radiation responding genes (Table 2). Notably, many genes that were down regulated by radiation were involved in metabolic processes, including the metabolisms of amino acids, carbohydrates and glycolysis. Other major groups of down-regulated genes were related to cell cycle, cytoskeleton and chromosomal replication and separation.

**Table 1:** Verification of RNA-seq transcript changes with RT-PCR.

Gene Name	RNA-seq^a^	RT-PCR^b^
ORF04957 DNA polymerase eta subunit	UD^c^	210.0
ORF07449 Sugar transporter	17	14
ORF07093 NRPS like enzyme	23	46
ORF01314 catalase A	6.3	16
ORF04811 phosphatidate phosphatase	17	UD^c^
ORF07066 fatty acid synthase subunit alpha	5	5
ORF02862 phytoene dehydrogenase	5	2
ORF02863 phytoene synthase	3.7	2.3
ORF04993 hypothetical protein	21	5.8
ORF04704 glucose-repressible gene protein-related	17	10
ORF03345 tyrosinase	5	3.8
ORF05621 dehydrogenase/reductase	56	6
ORF08836 Fe-Mn family superoxide dismutase	2.83	2.81
ORF03884 50S ribosomal protein L24e	2.03	9.82
ORF03371 hypothetical protein	UD^c^	4.31
ORF06166 tRNA pseudouridine synthase A	1.42	1.27
ORF95981 cyclin-dependent kinase CDC28	0.13	0.06
ORF05330 cell division cycle protein CDC20	0.2	0.26
ORF03906 alanyl-tRNA synthetas	0.004	0.09
ORF08633 amidase	0.02	0.008
ORF05584 4-hydroxyphenylpyruvate dioxygenase	0.05	0.011
ORF05586 methylisocitrate lyase	0.03	0.005
ORF05409 beta-glucosidase	0.04	0.004
ORF08979 alpha-galactosidase	0.05	0.007

a. Transcript ratio between irradiated and non-irradiated in the wild type strain from RNA-seq data.

b. Transcript ratio between irradiated and non-irradiated in the wild type strain from qRT-PCR data. qRT-PCR Ct values were derived from averages of duplicates from two independent biological samples.

c. Undivisible duo to no transcript was detected from non-irradiated samples.

### Melanin Affects Cellular Response to Ionizing Radiation by up-regulating Transporter Genes

**Figure 4:**
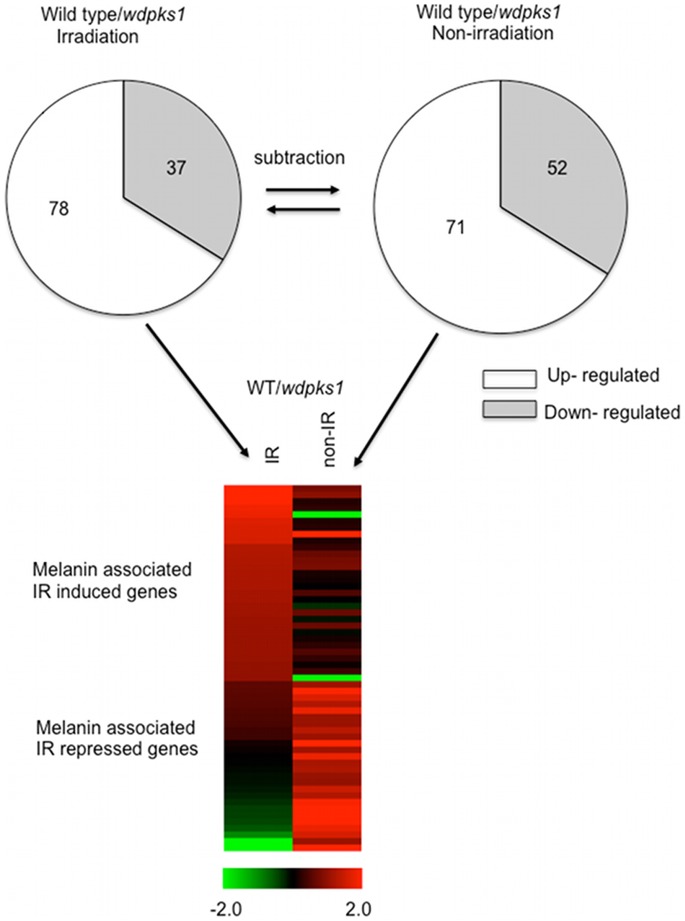
Expression profiles of radiation responding genes associated with melanin. First, differentially expressed genes with >2 fold changes when the wild type was compared with the *wdpks1* mutant in the presence of and absence of ionizing radiation were identified as shown in two pie charts (irradiation and non-irradiation group). Overlapped genes between these two groups were then removed from the irradiation group. Remaining genes represent those regulated by ionizing radiation and also associated with melanin, from which a heatmap was generated. Each column represents the radiation condition (IR: ionizing radiation; non-IR: no ionizing radiation). Each row represents log2 ratio of transcripts between the wild type and the *wdpks1* mutant for a single gene. Genes were ordered based on level of expression changes in the column IR.

In the scatter plot analysis (Figure 3A), a number of outliers (as shown by red and green colored spots) were noted, which were indicative of differentially expressed genes specific to either the melanized or albino strain. Therefore, before investigating the cellular response to radiation, we first determined whether melanin affected the gene expression profiling in the absence of irradiation. The analysis revealed that 201 genes were differentially expressed in the wild type compared to those in the *wdpks1* mutant grown in minimal media (FDR<0.001), suggesting that deletion of the *WdPKS1* gene not only inhibited melanin biosynthesis but also affected the expression of 2% of its total genes. Transcripts of 123 genes were changed at least 2 fold (71 induced and 52 repressed) (Figure 4), approximately half of which encoded proteins with unknown functions. Also, of the genes more expressed in the wild type, 25 that were assigned with KEGG functions mainly contributed to the metabolism of secondary metabolites (Table S2). Furthermore, comparison of the differential gene expression of the wild-type strain relative to that of the *wdpks1* mutant in the presence of low dose ionizing radiation showed that, of 345 differentially expressed genes, only 115 genes were changed by at least 2 fold (78 induced and 37 repressed) (Figure 4). Of those 115 genes, 58 genes were found to be regulated even in the absence of the radiation and were subsequently removed. The remaining 57 genes reflected radiation responding genes only associated with the presence of melanin (Figure 4). Among them, only 25 genes encoded proteins with known functions identified by pfam (Table S3). Interestingly, half of the genes induced in the wild type encoded transporter proteins involving transporting sugars, amino acids and other metabolites, whereas most of the remaining induced genes encoded oxidoreductases, transcription regulators and heat shock proteins.

**Table 2:** KEGG functions of regulated genes with >2-fold changes.

Functions	wild type			*wdpks1*		
	Total	Up	Down	Total	Up	Down
**Down regulation**						
Amino acid metabolism	57	7	50	61	6	55
Carbohydrate metabolism	18	1	17	17	1	16
Energy metabolism	5	1	3	3	1	2
Fatty acid metabolism	10	0	10	12	0	12
Galactose metabolism	6	1	5	6	1	5
Glycolysis/Gluconeogenesis	17	6	11	18	4	14
Cell cycle	12	1	11	17	1	16
Cytoskeleton proteins	8	0	8	9	0	9
Cellular process: Peroxisome	6	0	6	8	0	8
Replication and Repair, Chromosome	17	0	17	19	0	19
Translation, ribosome	1	0	1	9	0	9
Transporter: Major Facilitator Superfamily	7	2	5	8	2	6
**Up regulation**						
Fatty acid and lipid biosynthesis	17	11	6	17	11	6
Fructose and mannose metabolism	6	5	1	4	3	1
Folding, sorting and degradation	6	4	2	6	3	3
Glutathione metabolism	2	2	0	2	2	0
DNA repair and recombination	7	4	3	6	2	4
RNA polymerase	3	3	0	na	na	na
Starch and sucrose metabolism	3	3	0	4	3	1
Signaling molecules and interaction	9	5	4	8	5	3
Translation, ribosomal biogenesis	32	31	1	3	1	2
Translation, transfer RNA biogenesis	5	5	0	3	2	1
Transporter: Solute carrier family	7	5	2	5	2	3
Ion channels	7	5	2	7	5	2

### Ribosomal Biogenesis Gene Expression Under Low Dose Ionizing Radiation is Affected by the Presence of Melanin

**Figure 5:**
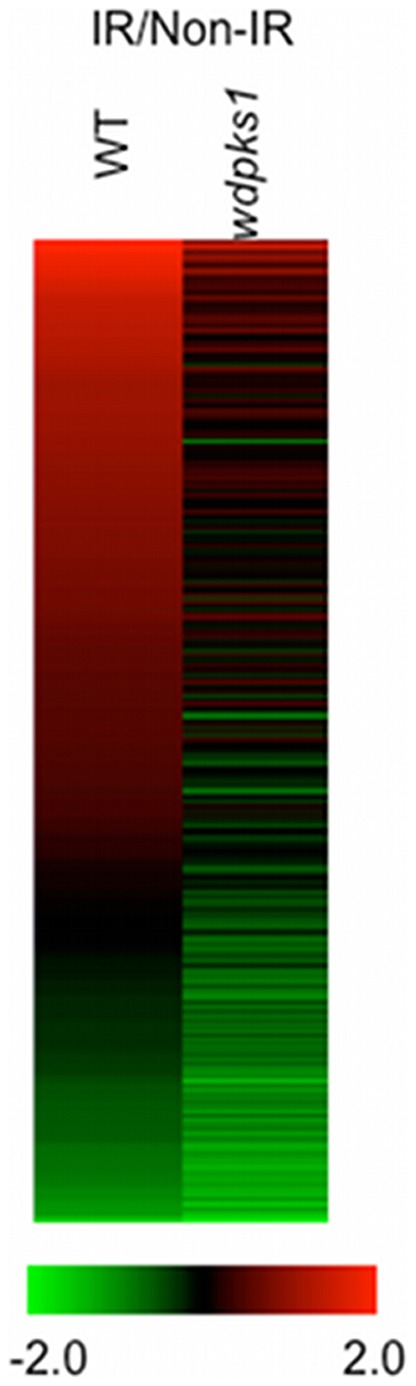
Expression profiles of RP/Ribi genes regulated by ionizing radiation in the wild type and *wdpks1* strains. Each column represents a strain. Each row represents log2 ratio of transcripts between irradiated and non-irradiated cells for a single gene. Genes were ordered based on level of expression changes in the wild type strain.

Gene expression patterns of irradiated wild type or *wdpks1* cells relative to their corresponding non-irradiated cells showed that the majority of radiation responding genes share consistent regulation patterns associated with KEGG functional categories with only one exception (Table 2). This involved a striking difference between the two strains in their regulation of genes encoding ribosomal protein (RP) and ribosomal biogenesis proteins (Ribi). In this category, 36 of 37 RP/Ribi genes were up-regulated more than 2 fold by ionizing radiation in the wild type, but only 3 RP/Ribi genes in *wdpks1* were up-regulated to the same level. To further examine the expression profile of all the RP/Ribi genes responding to radiation, we identified 87 RP genes and 200 Ribi genes in the *W. dermatitidis* genome to generate a gene expression heatmap (Figure 5). Contrary to the observation that RP/Ribi genes are usually down regulated under stress [22], [28], 101 RP/Ribi genes were up-reregulated (FDR <0.001) in the wild type strain, but only 17 were up-regulated (FDR <0.001) in the *wdpks1* mutant, under low dose ionizing radiation. This indicated that transcription of RP/Ribi genes in the wild type were influenced more robustly than those in the *wdpks1* mutant. For an even more detailed comparison, up-regulated RP/Ribi genes with more than 2-fold change in the wild type were compared with their corresponding genes in the *wdpks1* mutant (Table 3). These data revealed that those RP/Ribi genes in the *wdpks1* mutant were generally not affected by ionizing radiation. Therefore, this comparison indicated that the presence of melanin played a role in stimulating ribosomal biogenesis processes under ionizing radiation conditions. Additionally, genes encoding subunits of RNA polymerase I and III, which transcribe large rRNA, and tRNA, respectively, were all up-regulated more than 2 fold (Table 3) in the wild type.

**Table 3:** Up-regulated (2 fold) Ribi/RP genes.

Gene	Fold change		p-value	
	WT	*wdpks1*	WT	*wdpks1*
ORF06166 tRNA pseudouridine synthase A (701 aa)	8.18	1.03	1.27E-03	3.05E-02
ORF04237 cohesin complex subunit SCC1	3.73	1.62	1.20E-29	7.14E-09
ORF07051 SDA1 containing protein	3.72	2.41	6.14E-29	1.06E-21
ORF03892 RNA recognition motif	3.2	1.82	6.71E-20	4.53E-10
ORF02613 ribosomal RNA small subunit methyltransferase F	2.87	1.38	6.01E-21	1.85E-05
ORF08640 pyruvate dehydrogenase E1 component subunit beta	2.85	1.75	1.27E-20	2.53E-11
ORF02112 Cgr1 family	2.75	1.63	1.61E-11	8.09E-06
ORF05192 DNA-directed RNA polymerase I subunit A49	2.75	1.31	1.11E-16	5.12E-04
ORF04206 KRI-1 like family	2.7	1.24	2.09E-17	1.84E-03
ORF00131 Pin2-interacting protein X1	2.68	1.74	2.48E-16	9.76E-10
ORF05270 WD domain containing protein	2.62	1.57	1.63E-16	9.54E-08
ORF03747 U3 small nucleolar RNA-associated protein	2.58	1.19	2.46E-12	2.59E-02
ORF03955 RNA recognition motif	2.53	1.04	7.90E-01	4.28E-02
ORF04978 brix-domain ribosomal biogenesis protein	2.44	1.24	9.76E-14	3.72E-03
ORF08114 TRAUB (NUC102) domain containing protein	2.43	1.23	1.51E-13	4.25E-03
ORF04889 hypothetical protein	2.43	1.35	1.75E-12	4.92E-04
ORF01442 NUC153 containing protein	2.4	1.31	4.65E-14	3.54E-04
ORF04004 NAD-dependent histone deacetylase SIR2	2.36	1.15	1.44E-12	2.80E-02
ORF03300 tRNA-intron endonuclease	2.36	1.76	1.82E-14	6.79E-11
ORF08598 dimethyladenosine transferase	2.27	1.22	2.41E-12	4.90E-03
ORF07850 WD domain containing protein	2.23	1.47	5.86E-12	5.35E-06
ORF02803 WD domain containing protein	2.21	1.18	1.36E-12	9.40E-03
ORF05871 Nucleolar complex-associated protein	2.2	1.1	8.92E-12	5.97E-02
ORF02329 Rrp15p	2.19	1.17	6.02E-11	2.37E-02
ORF06261 ATP-dependent RNA helicase	2.19	1.14	1.70E-12	2.24E-02
ORF06916 18S rRNA biogenesis protein RCL1	2.17	1.31	8.20E-09	2.37E-03
ORF01125 ATP dependent RNA helicase	2.15	1.33	6.24E-11	3.02E-04
ORF00292 NUC091 containing protein	2.12	1.31	6.36E-12	2.03E-04
ORF05313 DNA-directed RNA polymerase I subunit A43	2.11	1.16	7.24E-11	1.78E-02
ORF01118 DNA-directed RNA polymerase II subunit H	2.11	1.45	1.89E-09	4.61E-05
ORF05393 PWI domain containing protein	2.1	1.61	6.41E-10	2.48E-07
ORF06122 Importin-beta N-terminal domain containing protein	2.1	1.24	7.77E-12	1.30E-03
ORF00494 DEAD/DEAH box helicase	2.08	0.97	6.48E-06	6.60E-01
ORF04715 hypothetical protein	2.06	1.18	6.78E-10	1.15E-02
ORF02347 periodic tryptophan protein	2.04	1.08	1.22E-10	7.96E-02
ORF07480 CSL zinc finger protein	2.03	1.29	9.83E-06	1.38E-02
ORF03884 50S ribosomal protein L24e	2.03	1.2	5.69E-10	8.07E-03
ORF04627 DEAD/DEAH box helicase	2.02	1.25	1.18E-09	1.74E-03
ORF07337 Exonuclease	2.02	1.42	9.00E-08	1.93E-04
ORF08644 Dip2/Utp12 Family	2.01	1.03	1.51E-10	2.10E-01
ORF01755 Ribosomal protein L1p/L10e family	2	1.09	2.29E-10	6.54E-02
ORF05245 Noc2p family	2	1.05	5.57E-10	1.40E-01

### Cell Cycle Genes are Repressed and DNA Translesion Synthesis Genes are Induced by Low Dose Ionizing Radiation

In contrast to the increasing number of cells that resulted from low dose radiation, all but one cell cycle and cytoskeleton genes identified in the functional annotation table were repressed at least 2 fold (Table 2). Furthermore, by comparison of cell cycle modules from *S. cerevisiae*, we identified 248 cell cycle related genes in *W. dermatitidis*. Aligning the gene expression values into a transcriptomic scatter plot indicated that the majority of cell cycle genes were down-regulated (Figure S3) and fifty of those cell cycle genes were significantly regulated as shown in Table 4. In contrast to some checkpoint genes, such as *MEC1*, *RAD9*, *RAD50* and *RAD53*, which are induced by DNA damage stress in *S. cerevisiae* [29], [30], their homologous genes in *W. dermatitidis* were repressed by the chronic and low dose of ionizing radiation in our study. A number of critical cell cycle genes, were down regulated as well. For example, transcription of the main cell cycle cyclin-dependent kinase *CDC28* gene, was repressed approximately 8 fold. Accordingly, expressions of *CDC20*, essential for exit from mitosis, deceased 5 fold, and *BUB1*, crucial for preventing cell cycle progression into anaphase in the presence of spindle damage, was down regulated 3 fold. Proteins encoded by these two genes were potential substrates of Cdc28. Another two protein kinase genes *MPS1* and *MAD*, required for spindle pole body duplication and mitotic spindle assembly, were repressed 2 to 3 fold as well. Furthermore, genes encoding cytoskeleton proteins (tublin, kinesin and actin), chromosome segregation proteins (histone, kinetochore) and DNA replication proteins were down-regulated by at least 2 fold. Finally, the *RNR1* transcript, which encodes the large subunit of ribonucleoside-diphosphate reductase and is required for the synthesis of dNTPs, was repressed 2 fold.

**Table 4:** Regulated cell cycle and DNA repair genes.

Gene names	Fold change		p-value	
	WT	*wdpks1*	WT	*wdpks1*
**ORF04957 DNA polymerase eta subunit RAD30/POL2**	**UD** *	**UD** *	**9.0E-124**	**5.9E-144**
**ORF02732 cryptochrome/6- photolyase**	**3.2**	**3.3**	**8.9E-25**	**1.6E-36**
**ORF04357 Cyclobutane pyrimidine dimer (CPD) photolyase**	**3.02**	**1.9**	**4.2E-20**	**3.7E-36**
ORF04004 NAD-dependent histone deacetylase SIR2	2.36	1.15	1.4E-12	2.8E-02
ORF02394 DNA mismatch repair protein MSH5	2.29	0.75	4.9E-10	4.0E-07
ORF09264 phosphate system cyclin PHO80	2.65	2.52	1.1E-18	3.0E-25
ORF02250 cell cycle protein MesJ	2.12	2.14	4.7E-12	3.4E-18
ORF05541 KilA-N domain (PF04388)	2.02	1.53	4.3E-11	5.6E-08
ORF04300 shikimate kinase	1.42	1.19	4.6E-02	1.1E-02
ORF07343 cell division control protein 48	0.69	0.59	3.1E-03	5.3E-06
**ORF07052 ataxia telangiectasia MEC1**	**0.68**	**0.74**	**2.2E-03**	**3.5E-02**
ORF02595 DNA polymerase epsilon catalytic subunit A	0.68	0.59	2.7E-03	5.7E-06
ORF01042 DNA polymerase delta subunit 1	0.66	0.56	1.1E-03	1.2E-06
ORF02342 replication factor C subunit 3/5	0.64	0.65	4.9E-04	7.5E-04
ORF02342 replication factor C subunit 3/5	0.64	0.65	4.9E-04	7.5E-04
**ORF03299 DNA repair protein RAD9**	**0.62**	**0.57**	**9.3E-05**	**1.3E-06**
ORF00781 G1/S-specific cyclin CLN3	0.62	0.6	8.9E-05	1.5E-05
**ORF04505 DNA repair protein RAD50**	**0.6**	**0.67**	**5.7E-05**	**2.2E-03**
ORF01328 DNA polymerase alpha subunit B	0.6	0.56	1.7E-04	1.2E-05
ORF04169 G2/mitotic-specific cyclin-B	0.6	0.48	3.9E-05	1.5E-10
ORF02265 cyclin H	0.59	0.56	2.3E-05	1.2E-06
ORF03155 anaphase-promoting complex component APC8	0.59	0.53	1.4E-05	3.8E-08
ORF06332 DNA replication regulator SLD2	0.59	0.52	3.0E-05	5.2E-08
ORF00177 cell division control protein 6 (cdc6)	0.58	0.45	7.2E-05	1.9E-10
ORF01910 cell division control protein 45	0.58	0.45	6.9E-05	7.8E-10
ORF02470 DNA-repair protein complementing XP-A cells	0.56	0.63	3.3E-06	2.0E-04
ORF03973 DNA repair protein rhp51	0.55	0.52	3.0E-06	4.0E-08
ORF06098 DNA polymerase iota subunit	0.56	0.61	3.6E-06	1.4E-04
ORF01786 G1/S-specific cyclin PLC2	0.51	0.36	3.7E-08	8.9E-21
ORF02802 DNA repair and recombination protein RAD52	0.51	0.44	1.5E-07	2.1E-11
**ORF06711 mitotic spindle assembly checkpoint protein MAD**	**0.5**	**0.38**	**2.8E-07**	**5.7E-14**
**ORF03847 ribonucleoside-diphosphate reductase large subunit RNR1**	**0.5**	**0.42**	**1.7E-08**	**8.1E-15**
ORF00901 ankyrin	0.5	0.44	3.5E-07	7.0E-10
ORF01628 tubulin gamma chain	0.5	0.46	1.9E-08	1.8E-11
ORF03590 replication fork protection complex subunit Tof1	0.45	0.38	1.2E-10	3.2E-17
ORF07079 replication fork protection complex subunit Csm3	0.44	0.39	3.9E-10	2.3E-14
**ORF07698 Ser/Thr/Tyr protein kinase RAD53**	**0.42**	**0.4**	**3.1E-13**	**6.6E-17**
ORF07994 DNA polymerase alpha subunit A	0.43	0.37	3.1E-11	6.6E-19
ORF08626 protein tyrosine kinase (PF07741)	0.4	0.43	1.1E-10	2.5E-09
ORF07609 Cyclin (PF08613)	0.4	0.44	7.2E-14	5.5E-13
ORF06670 GTPase activating protein	0.4	0.38	3.3E-14	1.3E-18
ORF00933 Telomeric single stranded DNA binding POT1	0.39	0.43	2.7E-14	1.3E-13
ORF04684 regulatory factor X, RfxX	0.38	0.26	5.9E-14	7.9E-31
ORF06442 cell division control protein 7	0.38	0.32	4.1E-12	1.4E-18
ORF04792 serine/threonine-protein kinase CHK1	0.38	0.35	1.2E-12	5.0E-16
ORF07861 cohesin complex subunit SCC1	0.38	0.6	3.7E-10	1.9E-03
ORF00159 kinesin family member 11	0.35	0.24	4.5E-14	7.6E-25
ORF04925 kinesin family member 18/19	0.35	0.26	1.3E-14	8.4E-26
ORF04792 serine/threonine-protein kinase Chk1	0.35	0.35	1.2E-12	5.0E-16
ORF01579 condensin complex subunit 3 Ycg1	0.34	0.29	6.7E-15	1.8E-21
**ORF02021 Ser/Thr protein kinase MPS1**	**0.32**	**0.34**	**2.5E-12**	**2.0E-12**
**ORF02791 checkpoint serine/threonine-protein kinase BUB1**	**0.31**	**0.28**	**9.1E-18**	**1.9E-23**
ORF00797 G2/mitotic-specific cyclin 3/4	0.26	0.23	2.0E-22	6.9E-30
ORF06441 condensin complex subunit 1	0.25	0.22	1.1E-26	8.1E-36
**ORF05330 cell division cycle protein CDC20**	**0.2**	**0.24**	**2.6E-38**	**8.5E-38**
ORF04488 kinesin family member C1)	0.17	0.17	9.3E-28	3.3E-31
**ORF05981 cyclin-dependent kinase 1 CDC28**	**0.13**	**0.14**	**5.0E-54**	**1.3E-62**
ORF04788 myosin heavy chain	0.11	0.09	4.2E-44	5.9E-59

Genes described in the text were in bold.

* Undivisible duo to no transcript was detected from non-irradiated samples.

To examine whether the reduced transcripts of cell cycle genes were related to the progression of the cell cycle itself, we investigated the change in DNA content by analyzing synchronized cells of the wild type strain. Starved cells were sorted by a fluorescence activated cell sorter (FACS) according to their sizes and two populations of cells corresponding to un-budded and budded cells, respectively, were obtained and subjected to 0.03R/hr radiation. Comparison of the DNA profiles between the non-irradiated and irradiated populations clearly showed that, during the first 24 hours, 32% of the irradiated cells entered S or G2 phase proceeded from un-budded cells and only 9% of the non-irradiated cells entered the same phase (Figure S4). On the other hand, from the population of budded cells, 66% of the irradiated cells finished mitosis and were arrested in G1 phase, but only 32.5% of the non-irradiated cells completed the cell cycle (Figure S4) during the first 24 hours. This finding suggested that the low dose of ionizing radiation might accelerate progression of the cell cycle by reducing transition lengths of both G1->G2 and G2->G1.

Interestingly, only a few up-regulated genes with more than a two-fold change were found to be involved in DNA translesion repair. Of particular interest was *RAD30*, which encodes a DNA polymerase eta (Pol eta), involves translesion synthesis (TLS) and allows the bypass of a variety of DNA lesions caused by oxidative damage in a largely error-free manner [31]. While the *RAD30* transcript in *W. dermatitidis* was not detected in non-irradiated cells with qRT-PCR, it was substantially induced in irradiated cells (Table 1), a result that agrees well with the report that *RAD30* transcription is induced in response to UV radiation in *S. cerevisiae* [32]. Another intriguing finding is that two genes that encode cyclobutane pyrimidine dimer (CPD) photolyase and cryptochrome/6-4 photolyase, respectively, were both induced 3 fold by the low dose ionizing radiation.

### Ionizing Radiation Elevates the Expression of Genes Involved in Membrane Fluidity and Water Transport

As shown in Table 2, the expression of genes for metabolic processes, such as metabolisms of amino acids, carbohydrates, energy and fatty acids were all down regulated by the ionizing radiation. However, it is interesting to note that many of the genes encoding enzymes for fatty acid and lipid biosynthesis were up-regulated (Table 5). Further sequence analysis indicated that those induced fatty acid biosynthesis genes were mainly involved in the synthesis of un-saturated fatty acids. Particularly, high expression of ▵^9^-desaturase (OLE1) and ▵^12^-desaturase (ODE12) genes suggested that increased desaturation of the fatty acids in cell membranes was induced by ionizing radiation. Moreover, a group of genes involved in metabolism of glycerophospholipids, the main structural component of cellular and organelle membranes, were highly induced by radiation. In *S. cerevisiae*, phosphatide phosphatase (PAP) plays a major role in the synthesis of phospholipids and triacylglycerols (TA), which is the major storage component for fatty acids and energy [33]. One PAP gene, homolog of DPP1 in yeast, was induced more than 17 fold in RNA-seq data in both the wild type and *wdpks1* mutant. In non-irradiated cells, expression of this gene was hardly detected by qRT-PCR but greatly increased in irradiated cells (Table 1). Coincident to these findings, another gene ORF03055 encoding a homolog of YOL002c of S. *cerevisiae*, was dramatically induced by radiation 25 and 9 fold in the wild type and the *wdpks1* mutant, respectively. YOL002c protein plays a key role in the regulation of lipid and phosphate metabolisms [34]. Interestingly, we found a number of genes encoding aquoporins and aquoglyceroporins were induced more than 2 fold by ionizing radiation. These two types of proteins channel water, small uncharged molecules and even H_2_O_2_ across the membranes so as to mediate nutrient uptake, stress regulation (eg. ROS stress) and other processes [35].

**Table 5:** Regulation of genes encoding phospholipids, fatty acids and transmembrane proteins.

Gene name	Fold change p-value			
	WT *wdpks1* WT *wdpks1*			
ORF03055 haemolysin-III family protein YOL002C	25.1	9.4	1.5E-105	1.4E-86
**ORF04811 phosphatidate phosphatase DPP1**	**17.12**	**22.14**	4.2E-113	9.1E-180
**ORF07950 phosphatidylserine decarboxylase**	**4.7**	**6.24**	2.1E-38	2.0E-67
**ORF00795 dihydroxyacetone kinase**	**4.48**	**3.34**	1.8E-40	1.6E-40
**ORF07729 phosphatidylserine decarboxylase**	**4.39**	**4.34**	6.8E-38	5.7E-53
**ORF09108 phospholipase A2**	**2.32**	**2.15**	1.7E-14	1.1E-18
**ORF08008 glycerophosphoryl diester phosphodiesterase**	**0.49**	**0.3**	8.4E-09	4.0E-27
**ORF08964 glycerol kinase**	**0.22**	**0.19**	5.9E-33	5.9E-49
*ORF08270 3-oxoacyl-[acyl-carrier protein] reductase*	*2.72*	*2.14*	3.5E-01	4.6E-09
*ORF07065 fatty acid synthase subunit beta FAS2*	*7.58*	*4.31*	3.9E-67	2.1E-56
*ORF07066 fatty acid synthase subunit alpha FAS1*	*5.19*	*3.41*	1.9E-47	5.5E-42
*ORF06163 Acetyl-CoA carboxylase ACC1*	*4.93*	*4.01*	8.9E-45	3.5E-51
*ORF09212 omega-6 fatty acid desaturase ODE12*	*3.34*	*2.64*	1.4E-27	5.6E-28
*ORF02574 acyl-CoA desaturase OLE1*	*4.1*	*2.66*	5.7E-36	5.5E-29
ORF03130 aquaglyceroporin like protein	5.91	4.8	3.3E-49	9.1E-56
ORF05178 aquaglyceroporin like protein	4.13	4.26	8.9E-31	3.4E-42
ORF07466 aquaporin related protein	3.36	5.14	2.4E-22	1.3E-48
ORF08570 aquaporin related protein	3.03	5.94	1.8E-23	3.8E-75
ORF04985 aquaglyceroporin like protein	2.5	2.72	6.2E-16	6.9E-26

Genes encoding glycerophospholipid metabolism were in bold and genes encoding fatty acid biosynthesis were in italic.

### Up-regulation of Antioxidant Genes Corresponds with Increased Survivability of W. dermatididis Cells Grown Under Low Dose Ionizing Radiation

**Figure 6:**
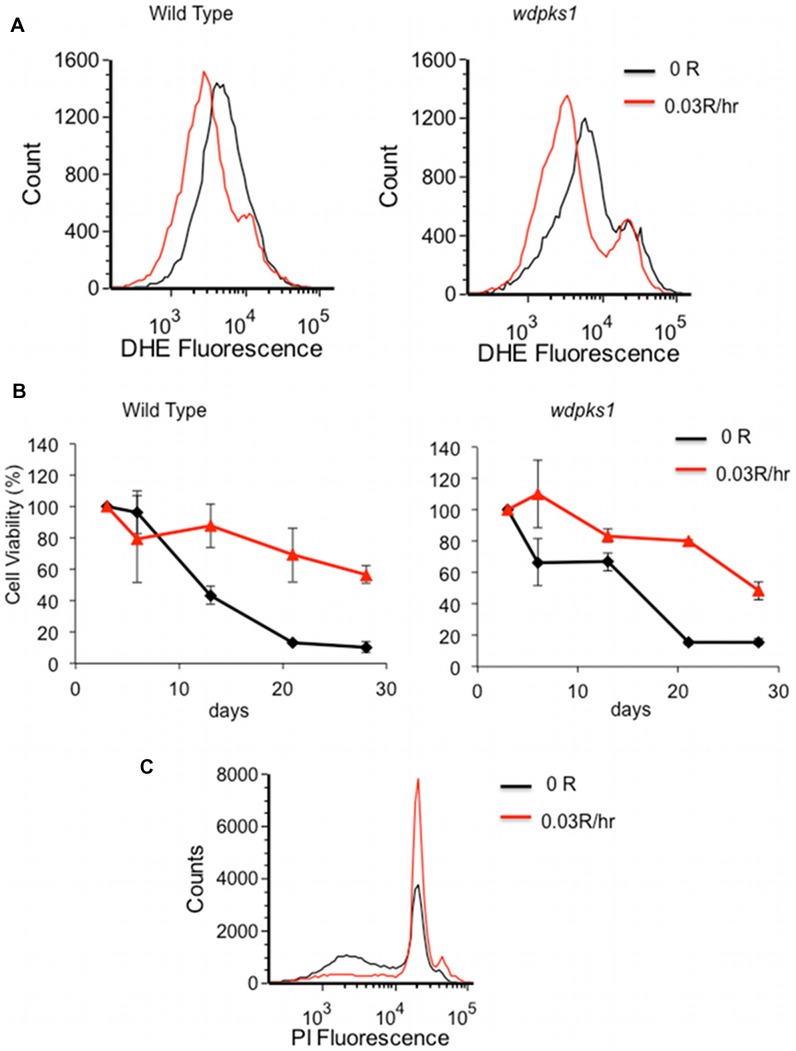
Low dose of ionizing radiation reduces ROS and increases survivability of *W. dermatitidis*. (A) Flow cytometry measurement of superoxide anions O_2_^−^ using the probe dihydroethidium (DHE) in the wild and *wdpks1* mutant cells exposed with 0.03R/hr ionizing radiation for 48 hours. The graph is a representative image from three independent experiments. (B) Long term effect of ionizing radiation (0.03r/hr) on viabilities of the wild type and *wdpks1* mutant. The percentage of CFUs was averaged from three independent experiments. (C) Flow cytometry analysis of DNA of the wild type cells grown in the absence and presence of ionizing radiation (0.03R/hr) for four weeks.

It is known that oxidative stresses, such as ionizing radiation, results in activation of DNA repair and antioxidant defense systems. We focused on the expression of antioxidant genes (Table 6). Two genes coding for glutathione S-transferases were induced ∼2.5 fold. Most notable were a gene encoding the cytosolic catalases (*CTT1*), which was induced up to 6.4 fold, and another gene encoding a mitochondrial Mn-dependent superoxide dismutase (*SOD2*), which was up regulated to 2.8 fold in both the wild type and *wdpks*1 mutant (Table 6). These two enzymes are known to defend against reactive oxygen species (ROS). To determine whether the induction of these genes was, in fact, correlated with reduced ROS levels, we measured changes of intracellular superoxide anions O_2_^−^ in non-irradiated and irradiated stationary phase cells using the probe dihydroethidium (DHE). This probe is non-fluorescent until it is oxidized by O_2_^−^ inside the cell. The results showed that the intracellular O_2_^−^ levels decreased in cells irradiated with 0.03 R/hr doses for 48 hours (Figure 6A). Since ROS has a damaging effect on cells, the reduced ROS level caused by the low dose of ionizing radiation prompted us to ask whether this level of radiation could increase the survivability of *W. dermatitidis*. To test this possibility, both the wild type and the *wdpks1* mutant were grown in minimal media and constantly irradiated with 0.03 R/hr of ionizing radiation for one month. Cell viability, as measured as colony forming units (CFU), showed that less than 20% of non-irradiated cells survived in the nutrient poor media, but irradiation improved cell viability by greater than 50% after 21 days (Figure 6B). Furthermore, when the same volumes of 4-week old cells stained with PI were analyzed with flow cytometry (Figure 6C), the DNA profiles indicated that the majority of cells were arrested in G0/G1 phase. Notably, the non-irradiated cells had half as many G0/G1 counts and a considerable amount of degraded DNA compared to irradiated cells. These findings suggested that *W. dermatitidis* was able to adapt to the low dose of ionizing radiation to enhance its viability by reducing ROS with up-regulation of genes encoding antioxidant enzymes.

**Table 6:** Antioxidant gene regulation.

Gene name	Fold change p-value			
	WT *wdpks1* WT *wdpks1*			
ORF06208 glutathione S-transferase	2.54	3.46	2.6E-17	9.2E-42
ORF04629 glutathione S-transferase)	2.25	2.16	1.9E-13	4.5E-18
ORF01314 catalase A CTT1	6.43	7.29	3.0E-58	1.1E-92
ORF08836 Fe-Mn family superoxide dismutase SOD2	2.83	2.53	1.8E-21	7.1E-26
ORF09198 geranylgeranyl pyrophosphate synthetase CrtE	0.57	0.64	5.0E-06	3.4E-04
ORF02862 phytoene dehydrogenase CrtI	4.93	4.36	3.0E-43	1.7E-53
ORF02863 phytoene synthase CrtYB	3.75	4.01	8.8E-32	1.8E-49
ORF02864 carotenoid cleavage dioxygenase	1.29	1.47	9.3E-03	2.1E-06
ORF02865 fumarate reductase flavoprotein subunit	3.17	2.64	1.3E-23	3.0E-25
ORF02866 Ca2+-transporting ATPase	2.11	1.95	5.2E-12	3.0E-15
ORF07092 XPG1-region domain protein	5.38	4.28	5.9E-47	1.1E-51
ORF07093 a-aminoadipate reductase	23.29	16.24	3.4E-136	4.4E-161
ORF07094 Fungal transcriptional factor	3.41	2.77	4.2E-28	5.6E-30
ORF07095 aldehyde reductase I (ARI)	15.5	9.12	6.6E-110	6.5E-112

### Ionizing Radiation Induces Gene Expression and Production of Carotenoid Biosynthesis

**Figure 7:**
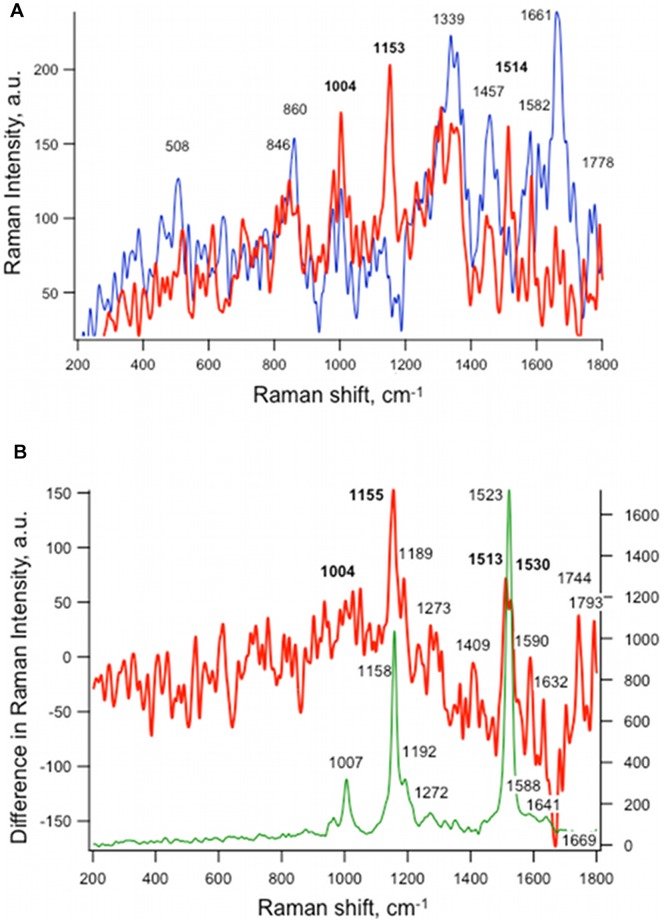
Low dose of ionizing radiation increases production of carotenoid in *W. dermatitidis*. (A) Raman spectra of individual *wdpks1* cells not irradiated (blue) and irradiated with 0.03R/hr radiation for 48 hr. (B) Subtracted Raman spectra between irradiated and non-irradiated cells. β-carotene in *Synechococcus* cells (green) is shown for the reference.

A four-gene cluster that includes genes encoding phytoene dehydrogenase and phytoene synthase, which are involved in carotenoid biosynthesis, were found to be up-regulated under low dose radiation (Table 6). Carotenoids are polyisoprenoid compounds responsible for the pigmentation of a number of fungi and play some role in radiation protection. Carotenoid synthesis has been shown to require photoinduction in *W. dermatitidis* [36]. To test whether high expression of carotenoid biosynthesis genes correlated with its production, we measured carotenoid components in single cells from both non-irradiated and irradiated samples using Raman spectroscopy. In order to exclude interference from the melanin pigment associated with the wild-type strain, this analysis was only performed with the *wdpks1* mutant cells that had been exposed to 0.03R/hr radiation for 48 hours. The results showed that the radiation induced a substantial increase in the intensity of bands at 1155, 1513–1530, 1744 and 1793 cm^−1^ (Figure 7A and B). Among them, the first two corresponded well with the absorption spectrum of β-carotene in cyanobacteria (Figure 7B). Moreover, increases in other (minor) bands specific for carotenoids (1192, 1272, 1588 and 1641) were also observed. For a better comparison, a spectrum of the non-irradiated *wdpsk1* cells was subtracted from that of the irradiated cells (Figure 7B). This demonstrated that the carotenoid bands at 1155 and 1523 cm^−1^ increased considerably, reflecting that a substantial production of carotenoid was induced by the ionizing radiation. However, no genes associated with the DHN melanin biosynthesis pathway showed significant regulation by the radiation, which agreed with the report that melanin in *W. dermatitidis* is constitutively synthesized (personal communication with Dr. Paul J. Szaniszlo).

## Discussion

In this study, we focused on investigating the molecular and cellular mechanisms of the black yeast *W. dermatitidis* and its melanin-defective mutant *wdpks1* in response to low doses of ionizing radiation. Overall, transcriptomic changes in the wild type and *wdpks1* mutant responding to the radiation, respectively, were generally similar as shown in Figure 3A, indicating that the transcriptional response to the low dose of ionizing radiation is mainly determined by cellular components other than melanin. However, our expression data still demonstrated that melanin played two roles upon ionizing radiation. First, in the presence of melanin, many transporter genes were up-regulated by radiation, which might help the melanized cells take up more nutrients (carbons and amino acids) and export toxic metabolites generated by the radiation stress. Second, the presence of melanin apparently affected expression of genes involved in the ribosomal biogenesis. In a growing cell, approximately 95% of total transcription and a large portion of total cellular energy are dedicated to ribosome biogenesis [37], [38], so up-regulation of those genes in the irradiated wild type cells in the carbon limited media caused us to speculate that low dose ionizing radiation, with the help of melanin, may stimulate ribosomal biogenesis machinery in the wild-type cells. This second possibility seems to support the intriguing hypothesis proposed by Dadachova et al [6] that melanin is able to harness ionizing radiation for metabolic energy. This hypothesis was further confirmed by the recent observation that three forms of radiation (visible light, UV light and gamma radiation) led to a reduction in the ATP levels only in melanized *C. neoformans* cells [39], which reflected that melanized cells might consume more energy when they were irradiated. The effect of melanin on energy conversion, however, is so subtle that further detailed investigations, such as the quantification of cellular protein and metabolic energy in single cells, are needed to prove this hypothesis. Unfortunately, due to our limited access to the radiation facility that is regulated by NRL, we were not able to measure the growth curves in even greater details. Nonetheless, we consistently demonstrated an increased number of *wdpks1* cells when exposed to ionizing radiation, a result in contrast to the previously reported finding that the growth of the same albino strain was not affected by radiation [6]. However, at least two possibilities may have accounted for this discrepancy: 1. Because the *wdpks1* mutant cells tend to aggregate and form multiple buds, these phenotypes might affect the accuracy of the CFU measurement; 2. Dadachova’s experiments were conducted under 0.005R/hr (0.05mGy/hr) dose of radiation created by a ^188^Re/^188^W generator. We irradiated cells with the dose of a 0.03R/hr in a ^137^Caesium field. Perhaps, the different energy emitted from those two types of isotopes caused different effects on cell growth. However, whereas in the former work, the albino strains did not show statistically significant growth changes under radiation, a trend toward slightly enhanced growth at late stage was apparent, particularly in non-melaninzed *C. neoformans* H99 strain [6]. Therefore, apart from melanin, we suggest that there may be other mechanisms in *W. dermatitidis* that regulate cell growth in response to low dose ionizing radiation, which were reflected by very similar expression profiles and similar adaption features in the wild type and the *wdpks1* mutant in this study.

Considering the enhanced growth of *W. dermatitidis* cultured under the low dose ionizing radiation used in our studies, it was surprising to see that the majority of cell cycle and DNA repair genes were down-regulated. The chronic low dose ionizing radiation and static growth in our study made it difficult to draw comparisons with the results of the transcriptomic studies in yeast models *S. cerevisiase* and *S. pombe*, which were generally performed with acute and higher lethal doses of ionizing radiation and in nutrient-rich growth conditions versus our nutrient-poor conditions. In former studies, typically DNA damage signature genes were induced by the high doses of ionizing radiation [7], [10] so as to repair damaged DNA and increase DNA replication irrespective of the resulting cell-cycle arrest point. However, in contrast to activation of checkpoint proteins in the DNA damage stresses, the majority of those same genes were repressed under the low dose ionizing radiation used in our study. The reduction in transcripts of cell cycle genes resulted in relatively shorter cell cycle, as is reflected in Figure S4. In budding yeast, a critical cell size is a prerequisite for progression through the cell cycle. Favorable growth conditions usually shortens G1 phase and coordinately enlarges cell size [40]. Our study demonstrated that both growth rates and cell sizes of *W. dermatitidis* were increased by low dose ionizing radiation. These phenotypes indicate that the low dose ionizing radiation was able to relieve the replicative stress that resulted from nutrient starvation in favor of the increased cell growth. Due to their relatively shorter cell cycle and faster growth rate, the irradiated cells depleted the nutrient quicker and therefore were arrested at G1 phase earlier than the non-irradiated cells, which could explain why irradiated cells had more un-budded phenotypes after the stationary phase as shown in Figure 2.

Mutagenic effect of ionizing radiation on the genome stability has been well investigated by dissecting double-strand breaks (DSB) in *S. cerevisiae*. The efficiency of random production of DSBs in yeast is estimated to be 0.083 DSBs/Mb/krad [41]. Considering 26 Mb genome size of haploid *W. dermatitidis* and total irradiation dosage (0.03 rad/hr for 48 hours), DSBs induced by the ionizing radiation in our study is calculated to be approximately 0.003/genome, which should not pose threats to genomic integrity. Although majority of DNA repairing genes were repressed (Table 4). it was surprising to find that three genes involved in DNA translesion synthesis (TLS) were strongly activated. DNA polymerase eta subunit (Pol η
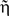
Rad30) is specialized to bypass *cis-syn* TT dimers caused by UV radiation [42], [43]. In humans, loss of Rad30 activity results in a cancer-prone syndrome known as xeroderma pigmentosum variant (XPV) [43]. Our study was the first report that *RAD30* gene was induced by ionizing radiation and also hinted that such radiation might cause single strand DNA lesion. The high expression of *RAD30* and the repression of genes encoding other polymerase subunits in response to the ionizing radiation make it more interesting to conduct the functional study so as to understand the physiological role of Polη
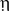
involved in ionizing radiation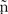 Coincidently, two other genes associated with UV response were also induced by the ionizing radiation. CPD photolyases repair UV-induced DNA damage by using a photorepairing mechanism and exist in almost all forms of life. Cryptochromes/6-4 photolyases are well-known blue-light photoreceptors that regulate a wide range of biological process in multicellular eukaryotes, but have only recently been found in plant fungi [44]–[46]. It is known that photolyase/cryptochrome family genes and carotenoid biosynthesis genes are regulated by UV and light in filamentous fungi [47] and DNA photolyases need to harvest light energy to repair DNA lesions [48]. However, our experiments were conducted in the absence of light and UV. Therefore, up-regulation of those genes in response to low dose ionizing radiation might suggest that those gene products are not only able to safeguard DNA but also able to potentially utilize ionizing radiation as energy for DNA repair.

Our RNA-seq data revealed that a variety of antioxidant genes were up regulated by low dose ionizing radiation, suggesting that *W. dermatitidis* responds to this oxidative stress by increasing production of free radical scavengers. This finding prompted us to investigate the biological functions of interesting targets, which is one of the main advantages for applying transcriptomic analysis to biological phenomena. Because ionizing radiation generates reactive oxygen species (ROS, eg., superoxide anion, hydrogen peroxide and hydroxyl radicals) from intracellular water, it results in a variety of oxidative damage to DNA, proteins, lipids and other cell components [49]. For this reason, biological systems have often evolved effective defense systems to cope with the oxidative environment. These include antioxidant enzymes, such as superoxide dismutase and catalase, and non-enzyme molecules, such glutathione, carotenoids and melanin [50]. Therefore, it is not surprising to see the up-regulation of *CTT1* and *SOD2* genes, indicating that *W. dermatitidis* cells are responding to oxidative stress in order to eliminate ROS induced by the low dose of ionizing radiation. The longstanding free radical theory of aging suggests that deleterious effects of free radical on macromolecules contribute to aging [51]. The induction of oxidative stress responses might thus promote longevity by protecting against reactive oxygen damages [52]. We suggest that the induction of genes encoding antioxidant enzymes, decreased ROS levels and extended lifespan resulting from chronic exposure to low dose radiation are consistent with this theory. Activation of oxidative stress and enzymatic DNA repair mechanisms, as found in this study are thought to enhance growth and extend lifespan and are reminiscent of radiation hormesis. This controversial hypothesis posits that chronic low dose ionizing radiation might be beneficial [53], but more experiments are needed to confirm this hypothesis. Similarly, we need to conduct more experiments dealing with carotenoids, whose synthesis was shown to require photoinduction in *W. dermatitidis* [36]. Our Raman spectral analysis of single cells subjected to ionizing radiation clearly demonstrated that the production of carotenoids in *W. dermatitidis* was due to induced gene expression of carotenoid biosynthesis genes. Therefore, we speculate that this inducible pigment might be playing some role in the sensing of and protection against ionizing radiation. Although the main pathway for carotenoid biosynthesis in fungi has been elucidated [54], the fundamental mechanism of the regulation of carotenogenesis in fungi is still not well understood [55]. Nonetheless, our data indicate that ionizing radiation is an activator of carotenogenesis, so further analysis of the transcriptomic data together with molecular approaches will allow us to find transcription factors that respond to ionizing radiation and interact with expression of carotenoid biosynthesis genes.

It was reported that the black yeast *Aureobasidium pullulans* adapts to salt stress by increasing expression of the fatty-acid-modifying enzymes, ▵^9^-desaturase (OLE1) and ▵^12^-desaturase (OLE12) [56]. Introduction of *cis* double bonds in positions 9 and 12 by these two enzymes increases the membrane fluidity. The high abundance of the desaturase mRNA seen at high salinities in *A. pullulans* was expected, but it is surprising to see the same abundance in the black yeast *W. dermatitidis* under low dose ionizing radiation. Possibly, the predicted decrease in the saturation levels of fatty acids, and therefore the increase in membrane permeability act as part of a stress-response mechanism to facilitate export of compatible solutes when they are no longer needed [57]. Recent studies in *S. cerevisiae* indicate that H_2_O_2_ challenge down-regulates the expression of genes encoding enzymes involved in lipid metabolism including those for fatty acid synthesis that lead to a reorganization of the plasma membrane and a lower permeability to H_2_O_2_ [58], [59]. However, our results revealed the opposite expression pattern for those genes, indicating increased permeability of H_2_O_2_ caused by ionizing radiation. Relevant to membrane structures, we found higher expression of a number of genes encoding aquaporins and aquaglyceroporins, the former of which is known in plants to facilitate the diffusion of H_2_O_2_ across membranes [60]. In yeast, expression of aquaporin genes are regulated depending on nutrients and osmotic stress in order to prevent cells from losing water and make them more resistant to harsh environmental conditions [35]. One study in human keratinocytes showed that UV irradiation induced the down-regulation of an aquaporin gene AQP3 and water permeability and resulted in dehydration of the skin, but adding antioxidant such as retinoic acid could induce AQP3 expression [61]. Therefore, our results suggest that *W. dermatitidis* cells appear capable of adapting to low dose ionizing radiation by enhancing membrane fluidity and further changing cellular metabolic activity. Taken together, we speculate that ionizing radiation might enhance the diffusion of ROS such as H_2_O_2_ by increasing permeability of plasma membranes and production of aquaporin/aquoglyceroporins to allow antioxidant enzymes to deactivate it efficiently.

In summary, this study highlights the use of high throughput transcriptome analysis to explore the molecular and cellular mechanisms exhibited by *W. dermatitidis* in response to low dose ionizing radiation. We revealed that ionizing radiation not only enhanced the growth of *W. dermatitidis* by affecting its cell cycle but also increased its survivability by inducing DNA translesion DNA synthesis and reducing the level of reactive oxygen species. Our findings indicate that participation of a variety of cellular components and pathways other than melanin allows this fungus to adapt to radiation environments for its survival and proliferation. Involvement of melanin in ribosomal biogenesis induced by ionizing radiation suggests the need for additional investigation to ascertain its role in the enhancement of cell growth. The knowledge gained from this study may benefit the assessment of the effect of long-term exposure to low doses of ionizing radiation to human health and aging and help in the development of strategies for radiation countermeasurement and radiotherapy.

## Supporting Information

Figure S1 (A) RNA-seq analysis of the wild type and the *wdpks1* strains without and with exposure of the ionizing radiation. (B) Correlation of RNA-seq data between duplicate samples.
(TIF)

Figure S2 Ionizing radiation (0.2 R/hr and 2 R/hr) has more significant effect on growth of the *wdpks1* mutant at the late stage. Non-irradiation (open bar), irradiation (hatched bar).
(TIF)

Figure S3 Differential gene expression profiles of cell cycle genes regulated by ionizing radiation in the wild type and *wdpks1* strains. Scatter plots of cell cycle genes (red spots) were integrated into the global gene expression plots (blue spots) as in Figure 3A.
(TIF)

Figure S4 FACS analysis of DNA of synchronized cells (un-budded and budded) in the absence (black) and presence of 0.03R/hr ionizing radiation (red) at 24th hr.
(TIF)

Table S1 Primer sequences for RT-PCR.
(DOCX)

Table S2 Differentially expressed genes affected by melanin in the absence of the ionizing radiation.
(DOCX)

Table S3 Differentially expressed genes affected by melanin under the low dose of ionizing radiation.
(DOCX)
