# Supplementary figures and images for: Adaptation of the Black Yeast Wangiella dermatitidis to Ionizing Radiation: Molecular and Cellular Mechanisms
Source: PLoS One. 2012 Nov 6;7(11):e48674. doi: 10.1371/journal.pone.0048674 (PMC3490873; doi:10.1371/journal.pone.0048674)

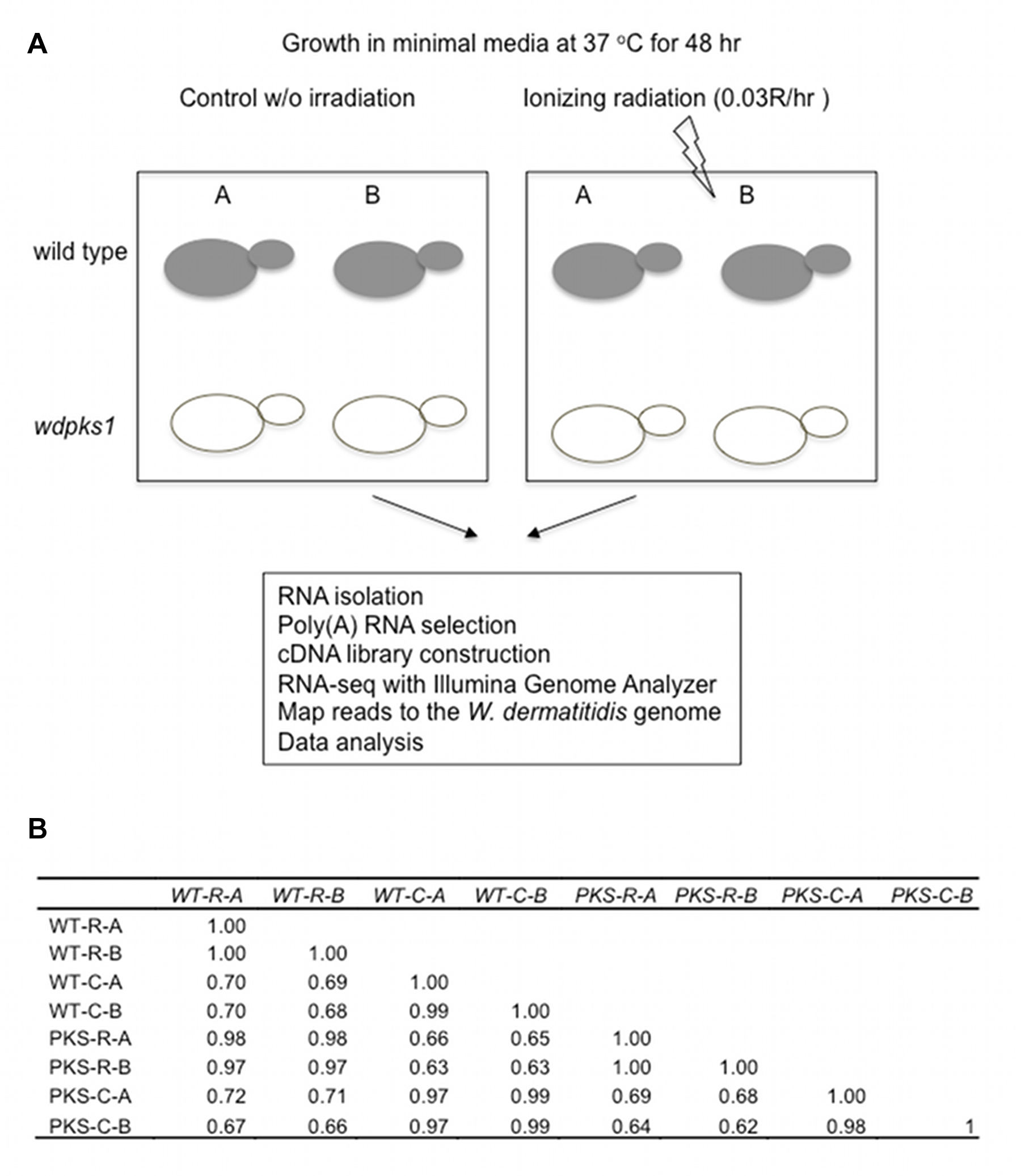

Supplement: Figure S2 — Ionizing radiation (0.2 R/hr and 2 R/hr) has more significant effect on growth of the wdpks1 mutant at the late stage. Non-irradiation (open bar), irradiation (hatched bar). (TIF) [file pone.0048674.s002.tif]

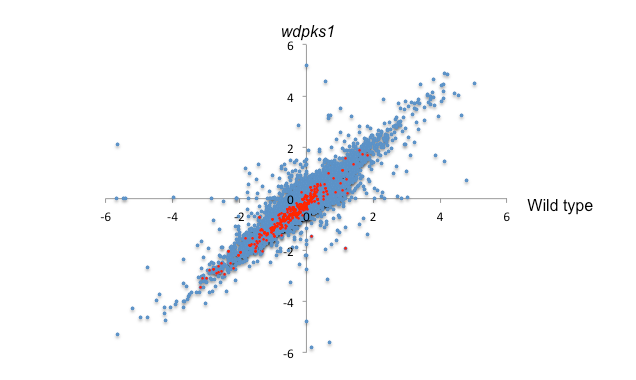

Supplement: Figure S3 — Differential gene expression profiles of cell cycle genes regulated by ionizing radiation in the wild type and wdpks1 strains. Scatter plots of cell cycle genes (red spots) were integrated into the global gene expression plots (blue spots) as in Figure 3A. (TIF) [file pone.0048674.s003.tif]

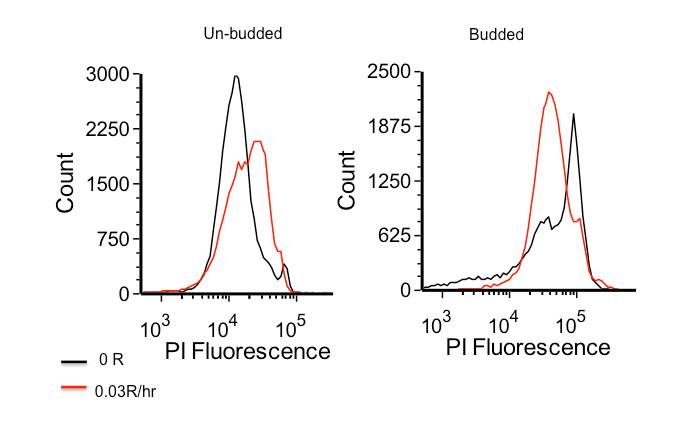

Supplement: Figure S4 — FACS analysis of DNA of synchronized cells (un-budded and budded) in the absence (black) and presence of 0.03R/hr ionizing radiation (red) at 24th hr. (TIF) [file pone.0048674.s004.tif]
