# Supplementary material for: Adaptation of the Black Yeast Wangiella dermatitidis to Ionizing Radiation: Molecular and Cellular Mechanisms
Source: PLoS One. 2012 Nov 6;7(11):e48674. doi: 10.1371/journal.pone.0048674 (PMC3490873; doi:10.1371/journal.pone.0048674)
Supplement: Table S1 — Primer sequences for RT-PCR. (DOCX) [file pone.0048674.s005.docx]

Table S1. Primer sequences for RT-PCR

| Primer name | Sequences (5' -> 3') |
| --- | --- |
| ORF01314_F | AGGCTCAGTTGTTCTGGAACTC |
| ORF01314_R | TGAGAGATCAATCTCGGTCAGA |
| ORF02862_F | ACCTTGCGGTCATCTACTGG |
| ORF02862_R | CGCAGACGAACTGTCTCAAA |
| ORF02863_F | TTGTCCCTTTTCCTGTCGAG |
| ORF02863_R | GTGAAGAACCGCTGATCCAT |
| ORF03345_F | GGTCTTGTCTCCGTTTCTGC |
| ORF03345_R | CGATACTGATCTCGGCCATT |
| ORF03371_F | TATGGACACGAAGAGAATGGTG |
| ORF03371_R | TGTGTGTCTGTGTCTGTCTTGG |
| ORF03884_F | GGAGGAGACTCAGTGGCAAG |
| ORF03884_R | ATGTCCATTTCCACCTCCAA |
| ORF03906_F | GTATTGCATCTTGGGCGTTT |
| ORF03906_R | TCGAGGTGTTGTGTGATGGT |
| ORF04704_F | TACGTCGGTGACAAGGTCAA |
| ORF04704_R | CAATCTTGTCAGAGGCAGCA |
| ORF04811_F | GTGGAGACCCTAACCAGATCAA |
| ORF04811_R | ACCTTCATCTTGGCATTGAGAT |
| ORF04957_F | CTACACCAAATCCCGAAGAGTC |
| ORF04957_R | CCTCAGGGTCTCCATTACTGTC |
| ORF04993_F | GGGGCAAACTATACCGTATGAA |
| ORF04993_R | CACGAACTCGATTGACTCTTTG |
| ORF05330_F | GGATGGAACAAGCACACCTT |
| ORF05330_R | ACTTCCGATGTGTGGGAGAC |
| ORF05409_F | CCTCTGGGGATCGTTTTACA |
| ORF05409_R | GCACGGAGACATTTGGTTTT |
| ORF05584_F | AGTATTTGCCCGTGGTGAAG |
| ORF05584_R | TCCTTGTCATCCACAGACCA |
| ORF05586_F | TGGTGTGGACGCAGTCTTTA |
| ORF05586_R | CTCCGTAAGACCACCCTCAA |
| ORF05621_F | ACCAGTGGCCTTGGATACTATG |
| ORF05621_R | ATTGTCCTGCTTTAGCTTTTGG |
| ORF05981_F | GGTGCCATATTCGCAGAGAT |
| ORF05981_R | TTGTCTTGAAGTCGGGGAAC |
| ORF06166_F | ACGAAGAACGAGAGCGATGT |
| ORF06166_R | TCTTTTCCGGGTTGATCTTG |
| ORF07066_F | GACTTCAGGTCCGCAGTCTC |
| ORF07066_R | TCGGATGTCCTCAACATCAA |
| ORF07093_F | CTAGGCTTCACCGTGCTTTACT |
| ORF07093_R | CCGAGACAGTCTTCTGAAAGGT |
| ORF07449_F | GGATCTTCATCGATTTGGTCTC |
| ORF07449_R | GAAATTGCGGAAGAAGATGAAC |
| ORF08633_F | AACGCAACAAGCTGTGTCAG |
| ORF08633_R | CTGGTTCTTGTCCACTGCAA |
| ORF08836_F | ATCGACACCACCTACGGAAG |
| ORF08836_R | TTGGTTGGCATATGTCTGGA |
| ORF08979_F | ATCACCACGGGAATGAATGT |
| ORF08979_R | GTGCCAGTCATCTTCCCATT |
