# Supplementary material for: Adaptation of the Black Yeast Wangiella dermatitidis to Ionizing Radiation: Molecular and Cellular Mechanisms
Source: PLoS One. 2012 Nov 6;7(11):e48674. doi: 10.1371/journal.pone.0048674 (PMC3490873; doi:10.1371/journal.pone.0048674)
Supplement: Table S2 — Differentially expressed genes affected by melanin in the absence of the ionizing radiation. (DOCX) [file pone.0048674.s006.docx]

Table S2: Differentially expressed genes affected by melanin in the absence of the ionizing radiation

| Genes | KEGG function |
| --- | --- |
| Up-regulated | |
| ORF07210 phenol 2-monooxygenase | Chlorocyclohexane and chlorobenzene degradation |
| ORF08562 phenol 2-monooxygenase | Chlorocyclohexane and chlorobenzene degradation |
| ORF02142 salicylate hydroxylase | Polycyclic aromatic hydrocarbon degradation |
| ORF07845 salicylate hydroxylase | Polycyclic aromatic hydrocarbon degradation |
| ORF05460 D-amino-acid oxidase | Penicillin and cephalosporin biosynthesis |
| ORF03575 hypothetical protein | Metabolism of xenobiotics by cytochrome P450 |
| ORF05266 nitrilase | Tryptophan metabolism |
| ORF02141 gentisate 1,2-dioxygenase | Tyrosine metabolism |
|  |  |
| Down-regulated | |
| ORF08197 homoserine O-acetyltransferase | Cysteine and methionine metabolism |
| ORF02385 MC family mitochondrial carrier protein | Solute carrier family |
| ORF01800 L-iditol 2-dehydrogenase | Fructose and mannose metabolism |
| ORF05015 hypothetical protein | Genetic Information Processing; Folding, Sorting and Degradation |
| ORF08598 dimethyladenosine transferase | Genetic Information Processing, Ribosome biogenesis |
| ORF03955 hypothetical protein | Genetic Information Processing, Ribosome biogenesis |
| ORF07668 hypothetical protein | Genetic Information Processing,Transfer RNA biogenesis |
| ORF06166 tRNA pseudouridine synthase A | Genetic Information Processing; Translation; Transfer RNA biogenesis |
| ORF02370 glycerol kinase | Glycerolipid metabolism  PPAR signaling pathway |
| ORF04364 glucarate dehydratase | Ascorbate and aldarate metabolism |
| ORF02813 F-box and WD-40 domain-containing protein 1/11 | Oocyte meiosis |
| ORF04237 cohesin complex subunit SCC1 | Ribosome biogenesis in eukaryotes |
| ORF03747 hypothetical protein | Ribosome biogenesis in eukaryotes |
| ORF05164 Cu2+-exporting ATPase | Unclassified; Metabolism; Energy metabolism |
| ORF00989 GPN-loop GTPase 3 like | Unclassified; Poorly Characterized; General function prediction only |
| ORF03055 hypothetical protein | Unclassified; Poorly Characterized; General function prediction only |
