# Supplementary material for: Adaptation of the Black Yeast Wangiella dermatitidis to Ionizing Radiation: Molecular and Cellular Mechanisms
Source: PLoS One. 2012 Nov 6;7(11):e48674. doi: 10.1371/journal.pone.0048674 (PMC3490873; doi:10.1371/journal.pone.0048674)
Supplement: Table S3 — Differentially expressed genes affected by melanin under the low dose of ionizing radiation. (DOCX) [file pone.0048674.s007.docx]

Table S3. Differentially expressed genes affected by melanin under the low dose of ionizing radiation

| Gene | Function |
| --- | --- |
| Induced^a^ | |
| ORF04221 | PF00011, Hsp20/alpha crystallin family |
| ORF09186 | PF00083, Sugar (and other) transporter |
| ORF03275 | PF00324, Amino acid permease |
| ORF07176 | PF00121, Triosephosphate isomerase |
| ORF04521 | PF04082, Fungal specific transcription factor domain |
| ORF04520 | PF00083, Sugar (and other) transporter |
| ORF02898 | PF00970, Oxidoreductase FAD-binding domain |
| ORF04436 | PF07690, Major Facilitator Superfamily |
| ORF04041 | PF07992, Pyridine nucleotide-disulphide oxidoreductase |
| ORF04403 | PF06609, Fungal trichothecene efflux pump (TRI12) |
| ORF02144 | PF04082, Fungal specific transcription factor domain |
| ORF07075 | PF08591, Ribonucleotide reductase inhibitor |
| ORF00234 | PF00324, Amino acid permease |
| ORF00092 | PF07690, Major Facilitator Superfamily |
| ORF05464 | PF07350, Protein of unknown function (DUF1479) |
| ORF04344 | PF03807, NADP oxidoreductase coenzyme F420-dependent |
| Repressed^b^ | |
| ORF07454 | PF01145, SPFH domain / Band 7 family |
| ORF05266 | PF00795, Carbon-nitrogen hydrolase |
| ORF02013 | PF06500, Alpha/beta hydrolase of unknown function (DUF1100) |
| ORF05460 | PF01266, FAD dependent oxidoreductase |
| ORF00144 | PF11905, Domain of unknown function (DUF3425) |
| ORF05643 | PF01073, 3-beta hydroxysteroid dehydrogenase/isomerase family |
| ORF06822 | PF00076, RNA recognition motif |
| ORF08262 | PF05704, Capsular polysaccharide synthesis protein |
| ORF03088 | PF02230, Phospholipase/Carboxylesterase |

^a^ Transcript ratio of WT/*wdpks1* > 2 under irradiation.

^b^ Transcript ratio of WT/*wdpks1* <0.5 under irradiation.
